# Millennium-old pathogenic Mendelian mutation discovery for multiple osteochondromas from a Gaelic Medieval graveyard

**DOI:** 10.1038/s41431-022-01219-2

**Published:** 2022-11-28

**Authors:** Iseult Jackson, Valeria Mattiangeli, Lara M Cassidy, Eileen Murphy, Daniel G Bradley

**Affiliations:** 1 Smurfit Institute of Genetics, Trinity College Dublin, Dublin 2, Ireland; 2 The SFI Centre for Research Training in Genomics Data Science, NUI Galway, Ireland; 3 Archaeology and Palaeoecology, School of Natural and Built Environment, Queen’s University Belfast, Belfast, Northern Ireland

## Abstract

Only a limited number of genetic diseases are diagnosable in archaeological individuals and none have had causal mutations identified in genome-wide screens. Two individuals from the Gaelic Irish Medieval burial ground of Ballyhanna, Co. Donegal, showed evidence of bone tumours consistent with the autosomal dominant condition multiple osteochondromas. Genome sequencing of the earlier individual uncovered a missense mutation in the second exon of *EXT1*, a specific lesion that has been identified in several modern patients. The later individual lacked this but displayed a novel frameshift mutation leading to a premature stop codon and loss of function in the same gene. These molecular confirmations of a paleopathological diagnosis within a single rural ancient context are surprisingly disjunct, given the observation of clusters of this disease in modern isolated populations and a *de novo* mutation rate of only 10%.

Cases of Mendelian genetic disease have been uncovered in the archaeological record, but diagnosis is restricted to osteologically visible conditions, with more commonly encountered conditions including achondroplasia, multiple epiphyseal dysplasia and Léri-Weill dyschondrosteosis (1).

Our study focuses on two adult male individuals (Sk197 and Sk331) buried in a Medieval graveyard at Ballyhanna, near the town of Ballyshannon in Co. Donegal, Ireland. Each displayed multiple bony tumours suggestive of multiple osteochondromas (MO), a rare, autosomal dominant bone condition, also known as hereditary multiple exostoses (2). Although these tumours are normally benign, the condition can result in limb deformity, reduced stature, compression of nerves, and, more rarely, malignancy(3). Surgical intervention is used to treat patients with severe osteochondromas, although those with asymptomatic MO do not require treatment (4).

The rural burial ground at Ballyhanna was associated with a small Medieval church, constructed after the middle of the 13th century AD, although it is possible that an earlier wooden church was present on the site. The land at that time would have been owned by a bishop and the estate lands would have been managed by an *erenagh* (estate manager). Radiocarbon dating showed that the earliest burials dated to the late 7th to early 8th century, but the vast majority of individuals were interred between AD 1200 and 1650, when the area around Ballyshannon was under the autonomous control of the Ó Domnaill Gaelic lords. As such, the graveyard at Ballyhanna can be considered to have essentially contained the remains of a Gaelic Medieval population. Those buried at Ballyhanna would have comprised the lower classes and included tenant farmers, labourers, merchants, artisans, clergy and the very poor(2).

Radiocarbon dating revealed that the two individuals were definitely not contemporaneous and were potentially separated by several hundred years. Of the two, Sk197 was the earlier individual (dated AD 689-975; UBA-11443) and was slightly older (30-40 years) when he died. While multiple osteochondromas were evident throughout his skeleton (Figure 1 a.), they were generally less pronounced than those evident in Sk331. Limb length discrepancy was present in his forearm bones, his sacro-iliac joints displayed ankylosis and he would have had genu valgum during life. Unlike Sk331, he was estimated to have been of roughly average height for the population (166.8 cm). Sk331 was dated to AD 1031-1260 (UBA-11442) (Figure S1; Table S1) and was the more severe case. He displayed extensive bilateral osteochondromas, both sessile and pedunculated in form, on most bones throughout his skeleton (Figure 1 b.). He also had a short stature compared to other adult males at Ballyhanna (158.3 cm), displayed a major deformity of his left forearm due to shortening of the ulna (Type 1)(5), had unequal bone lengths due to the lesions, as well as a range of orthopaedic deformities that affected his hips, knees and left ankle; all of which are consistent with this condition(5). He died as a young adult (18-25 years). Neither individual appears to have suffered from any tumours that progressed to malignancy(2).

In order to identify likely causative mutations, we used an unbiased genome wide approach involving shotgun sequencing and ancient DNA protocols (see SI). Petrous bone samples from each individual yielded intermediate levels of endogenous DNA preservation (Sk331: 12.2%; Sk197: 13.9%) and were sequenced to a mean depth of coverage of 4.2× and 5.1× respectively (see Table 1 and Table S2 for summary and sequencing statistics). Both samples had low contamination estimates for both the X and mitochondrial chromosomes (Table S3). Likely pathogenic mutations in *EXT1* were identified from an exome-wide scan in both individuals. All possible variants were initially filtered for quality (minimum allelic depth 3; maximum read depth twice the mean genomic coverage; minimum genotype quality 50) and predicted molecular impact using SnpEff and SnpSift (high or moderate impact)(6,7). All qualifying variants were heterozygous calls. Therefore, only variants in genes with a high probability of loss of function intolerance (pLI > 90%) according to gnomADv2.1.1 were retained, in order to filter out variants that were unlikely to cause a pathological phenotype in a heterozygous state(8). These variants were then assessed based on allele frequencies (<1% in gnomAD), *in silico* predicted pathogenicity (according to SIFT and Polyphen2) and publicly available data from ClinVar(8–11). Only one mutation in each individual had the level of support required by the American College of Medical Genetics guidelines to classify them as pathogenic (Figure 1; Table S4)(12).

The predicted pathogenic mutation identified in Sk197 is a missense mutation in the second exon of *EXT1* (NC_000008.10:g.118849385G>A; NP000118:p.Arg340Cys) (Figure 1c.). This specific mutation has been identified in at least three patients (ClinVar accession: VCV000002500.8), and different missense mutations at the same amino acid residue have been identified as pathogenic (R340G,L,H: accessions VCV000988576, VCV000002495, VCV000265129), as have substitutions at nearby residues. Arginine to Cysteine is a non-conservative amino acid substitution, and at this position has been demonstrated to disrupt EXT1/EXT2 complex activity, consistent with what is known about disease mechanism(13). Therefore, there are multiple lines of evidence supporting a pathogenic role for this mutation in Sk197: the same amino acid change as an established disease variant is observed; functional studies have shown a deleterious effect; this mutation is absent in population databases; computational evidence supports a damaging effect on the gene product; finally, the phenotype (MO) is highly specific for this gene.

Although the G>A mutation is not found in Sk331, a novel predicted pathogenic mutation is observed. This mutation is a C insertion within the first exon of *EXT1* (NC_000008.10:g.119122909_119122910insC; NP_000118:p.(Lys126Argfs*63)), resulting in a frameshift mutation and premature stop codon (Figure 1d.). This is a very severe mutation, resulting in a complete loss of the protein product of one copy of *EXT1* and is consistent with what is known about pathogenic mutations associated with this disease(3). Although this mutation has not been observed in modern patients (as reported in ClinVar; date accessed 11-02-2022), there are 6 frameshift variants predicted to be pathogenic within 50bp of this site, and 3 nonsense SNVs predicted to be pathogenic in this region (Table S5). Therefore, this is a null mutation where loss of function is a known mechanism of disease at a locus where there is modern clinical data supporting pathogenicity. Computational tools predict this variant to be deleterious. In sum, there is a high level of evidence to support the pathogenicity of this variant.

90% of cases of MO are caused by mutations in the exostosin genes (*EXT1* and *EXT2*), which are involved in heparan sulfate chain synthesis and assembly(3). These chains interact with a wide range of signalling molecules, and deficiencies in these interactions lead to altered signalling pathways (3). Most of these mutations are classified as inactivating mutations; they result in a complete loss of the protein product from the copy of the gene carrying the mutation(3). As *EXT1* and *EXT2* form a complex to carry out their molecular function, such single allele mutations in either gene cause a reduction in the quantity of functional complex and have physiological effect (for example, defects in lipid metabolism and clearance). However this is insufficient for tumour formation(3). MO is a dominant condition and it is thought that a complete somatic loss of EXT1/2 function is necessary for disease, which has been reported as being due to loss of heterozygosity or aneuploidy(3).

To date only stature phenotypes in recent historical samples have led to successful identification of causal Mendelian lesions; none with a genome-wide approach. For example, an 18th century skeleton of an extremely tall individual had sufficiently intact DNA for targeted PCR of a gigantism-associated locus(14). A pathological achondroplasia mutation in the FGFR3 gene has also been identified in 180-year old remains(15).

We used projection principal components analysis with modern northwest European populations to test affinities of the two Ballyhanna genomes (see SI Methods)(16–18). Both individuals fall at the overlap between Scottish and Irish samples, consistent with what we might expect for modern individuals from the northern part of Ireland(16) (Figure 2).

These men had different mitochondrial haplogroups but fell into the same clade of Y chromosome haplotypes, although Sk331 had a slightly more derived Y haplogroup (Table 1). Both grouped in the cluster R1b-M222, which is known to have its highest frequency in the same northwestern region in modern Ireland(19). Interestingly, from modern examination of surnames, the dominant Ó Domnaill Gaelic clan in this region would have been expected to display this haplotype(19).

Genome wide diploid genotypes were estimated for both individuals using imputation. For context, these were merged with a dataset of 78 Iron Age-Medieval Eurasian imputed genomes and phased (see SI Methods). This was used to test whether the Ballyhanna individuals shared recent ancestors, or exhibited shared haplotypes around either disease gene. They did not share IBD in excess of that observed among the reference dataset (Figure S2); these individuals were very unlikely to be related. The haplotypes around *EXT1* and *EXT2* were also visualised using haplostrips (Figure S3)(20) and those from Sk197 and 331 do not cluster together. This supports the conclusion that these two individuals had mutations of independent origin.

MO cases have been osteologically identified in the palaeopathological record from the Middle Bronze Age to the post-medieval period (5). Ten of these cases are isolated examples, but four countries have multiple individuals with MO: Gotland (Sweden) (n=2: mother and unborn infant); Jordan (n=3, of which two are broadly contemporaneous), England (n=3) and Ireland (n=4, including the 2 probands here)(5). The estimated incidence of this condition in modern individuals is approximately 1/50000, although higher incidences have been reported in the isolated population of the Chamorros of Guam (1/1000)(4). The *de novo* rate of mutation in this condition is low at 10%(4), and this clustering in modern, restricted populations strongly suggests founder effects typical of severe dominant genetic diseases. Such would have been expected within the same graveyard in the north-western corner of Gaelic Medieval Ireland, and therefore it is striking that our analyses demonstrate these two rare cases arise from separate mutational events.

## Supplementary Material

Supplemental tables

Supplemental text and figures

## Figures and Tables

**Figure 1:**
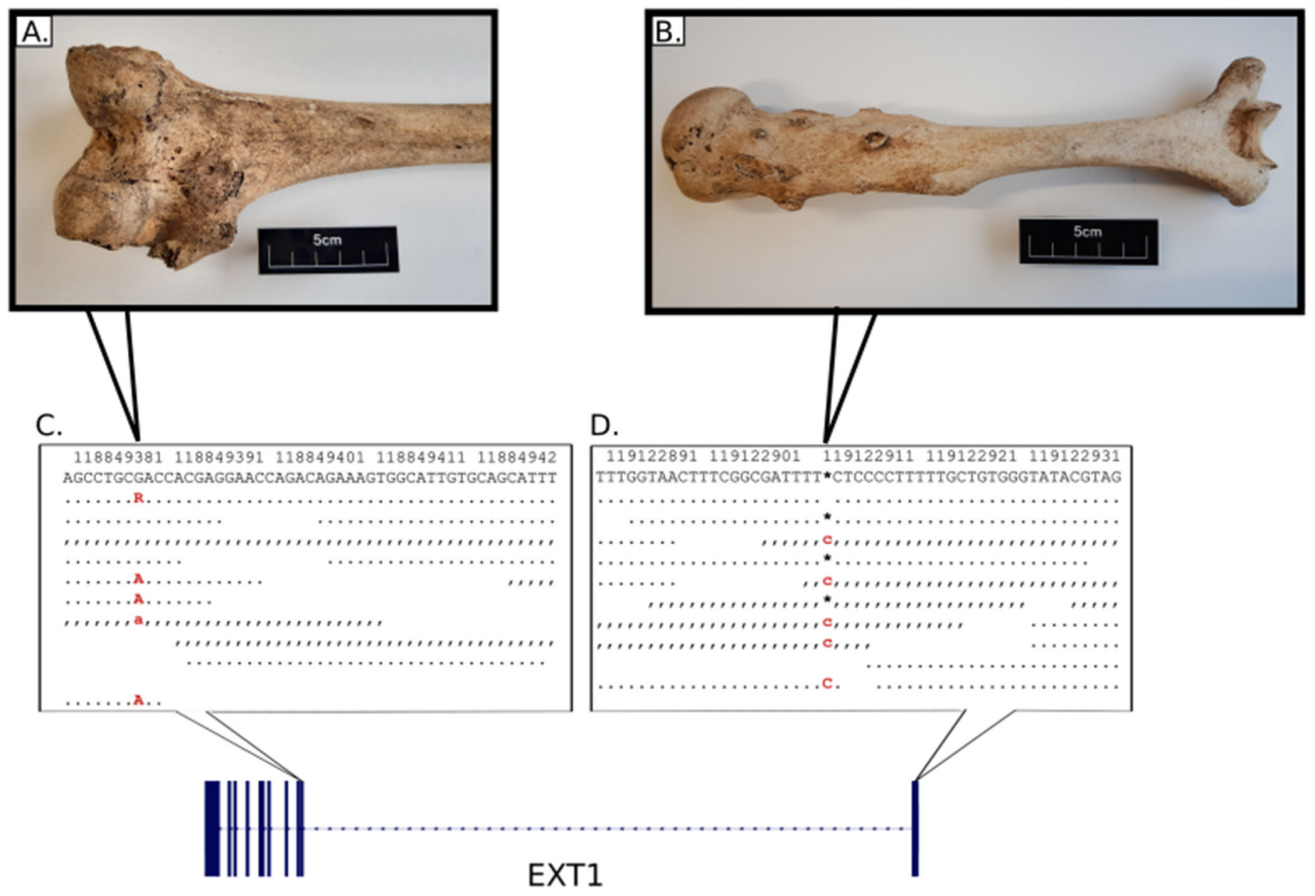
Examples of lesions and alignment of NGS data at putative disease variants. EXT1 gene structure with disease variants highlighted in red and bold typeface. First line: hs37d5 reference sequence. Second line: consensus for each individual. Dots: bases match reference on forward strand. Commas: bases match reference on reverse strand. R: consensus base is an A or G. A) Example of large sessile osteochondroma on the posterior surface of the distal end of Sk197's left femur. B) Example of sessile and pedunculated osteochondromas on the posterior surface of Sk331's left humerus. C) G>A transition in exon 2 of EXT1 in Sk197, resulting in Arginine to Cysteine mutation at the corresponding amino acid residue. D) C insertion in exon 1 of EXT1 in Sk331.

**Figure 2:**
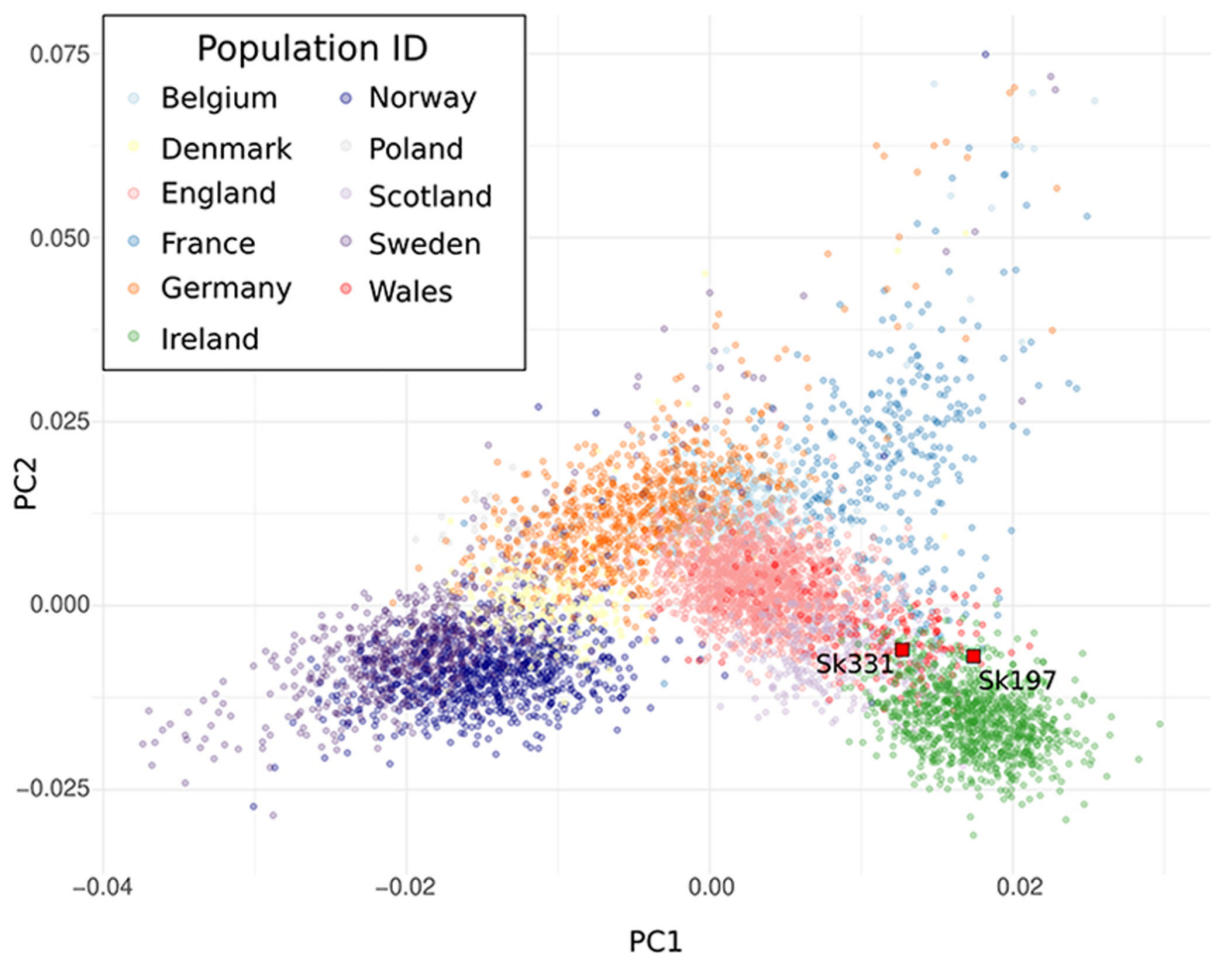
Principal Component Analysis of North-west European populations. Sk331 and Sk197 projected onto a plot of North-west European populations. Modern populations: Ireland (green); Scotland (lilac); Wales (red); England (pink); France (blue); Germany (orange); Belgium (pale blue); Norway (dark purple); Sweden (purple); Denmark (yellow); Poland (grey).

**Table 1:** Sequencing Summary. Summary of dates, coverage, sex and uniparental haplogroups assigned using MitoMaster and Phylotree 17 (mitochondrial) and ISOGG v.15.58 (Y). Both samples were dated twice (see Table S1 for details): both measures are reported here.

ID	Genomic Coverage	Sex	mtDNA Haplogroup	Y Haplogroup	Radiocarbon date (Measure A) (2 σ)	Radiocarbon date (Measure B) (2 σ)
Sk331	4.2×	XY	H13a1a	R1b1a1b1a1a2c1a1a1a1a1a3a (SNPs: M207; M343; M269; P312; S245; L21; M222; DF106; DF104; DF105; A259; A260)	AD 1158-1260	AD 1031-1211
Sk197	5.1×	XY	H4a1a2a	R1b1a1b1a1a2c1a1a1a1a1a1a (SNPs: M207; M343; M269; P312; S245; L21; M222; DF106; DF104; DF105)	AD 689-885	AD 774-975

## Data Availability

Raw FASTQ and aligned BAM files are available from the European Nucleotide Archive (ENA) under accession number PRJEB50653.

## References

[1] Lewis M, Buikstra JE (2019). Ortner’s Identification of Pathological Conditions in Human Skeletal Remains.

[2] McKenzie CJ, Murphy EM (2018). Life and Death in Medieval Gaelic Ireland: The Skeletons from Ballyhanna, Co Donegal.

[3] Pacifici M (2017). Hereditary Multiple Exostoses: New Insights into Pathogenesis, Clinical Complications and Potential Treatments. Curr Osteoporos Rep.

[4] Wuyts W, Schmale GA, Chansky HA, Raskind WH (2020). Hereditary Multiple Osteochondromas. GeneReviews®.

[5] Murphy EM, McKenzie CJ (2010). Multiple osteochondromas in the archaeological record: A global review. J Archaeol Sci.

[6] Cingolani P, Platts A, Wang LL, Coon M, Nguyen T, Wang L (2012). A program for annotating and predicting the effects of single nucleotide polymorphisms, SnpEff: SNPs in the genome of Drosophila melanogaster strain w1118; iso-2; iso-3. Fly.

[7] Cingolani P, Patel VM, Coon M, Nguyen T, Land SJ, Ruden DM (2012). Using Drosophila melanogaster as a Model for Genotoxic Chemical Mutational Studies with a New Program, SnpSift. Front Genet.

[8] Karczewski KJ, Francioli LC, Tiao G, Cummings BB, Alföldi J, Wang Q (2020). The mutational constraint spectrum quantified from variation in 141,456 humans. Nature.

[9] Kumar P, Henikoff S, Ng PC (2009). Predicting the effects of coding non-synonymous variants on protein function using the SIFT algorithm. Nat Protoc.

[10] Adzhubei I, Jordan DM, Sunyaev SR (2013). Predicting functional effect of human missense mutations using PolyPhen-2. Curr Protoc Hum Genet.

[11] Landrum MJ, Lee JM, Benson M, Brown GR, Chao C, Chitipiralla S (2018). ClinVar: improving access to variant interpretations and supporting evidence. Nucleic Acids Res.

[12] Richards S, Aziz N, Bale S, Bick D, Das S, Gastier-Foster J (2015). Standards and guidelines for the interpretation of sequence variants: A joint consensus recommendation of the American College of Medical Genetics and Genomics and the Association for Molecular Pathology. Genet Med.

[13] McCormick C, Duncan G, Goutsos KT, Tufaro F (2000). The putative tumor suppressors EXT1 and EXT2 form a stable complex that accumulates in the Golgi apparatus and catalyzes the synthesis of heparan sulfate. Proc Natl Acad Sci U S A.

[14] Chahal HS, Stals K, Unterländer M, Balding DJ, Thomas MG, Kumar AV (2011). AIP Mutation in Pituitary Adenomas in the 18th Century and Today. N Engl J Med.

[15] Boer LL, Naue J, de Rooy L, Oostra RJ (2017). Detection of G1138A Mutation of the FGFR3 Gene in Tooth Material from a 180-Year-Old Museological Achondroplastic Skeleton. Genes.

[16] Byrne RP, Martiniano R, Cassidy LM, Carrigan M, Hellenthal G, Hardiman O, Falush D (2018). Insular Celtic population structure and genomic footprints of migration. PLoS Genet.

[17] Leslie S, Winney B, Hellenthal G, Davison D, Boumertit A, Day T (2015). The fine-scale genetic structure of the British population. Nature.

[18] Sawcer S, Hellenthal G, Pirinen M, Spencer CCA, International Multiple Sclerosis Genetics Consortium, Wellcome Trust Case Control Consortium 2 (2011). Genetic risk and a primary role for cell-mediated immune mechanisms in multiple sclerosis. Nature.

[19] Moore LT, McEvoy B, Cape E, Simms K, Bradley DG (2006). A Y-Chromosome Signature of Hegemony in Gaelic Ireland. Am J Hum Genet.

[20] Marnetto D, Huerta-Sánchez E (2017). Haplostrips: revealing population structure through haplotype visualization. Methods Ecol Evol.

