# Supplementary material for: Millennium-old pathogenic Mendelian mutation discovery for multiple osteochondromas from a Gaelic Medieval graveyard
Source: Eur J Hum Genet. Author manuscript; Available in PMC 2023 Feb 9. (PMC9905557; doi:10.1038/s41431-022-01219-2)
Supplement: Supplemental text and figures [file EMS155735-supplement-Supplemental_text_and_figures.docx]

**Supplemental Information**

**Millennium-old pathogenic Mendelian mutation discovery for multiple osteochondromas from a Gaelic Medieval graveyard.**

Iseult Jackson^1,2^, Valeria Mattiangeli^1^, Lara M Cassidy^1^, Eileen Murphy^3^*, Daniel G Bradley^1^*

1. Smurfit Institute of Genetics, Trinity College Dublin, Dublin 2, Ireland

2. The SFI Centre for Research Training in Genomics Data Science, NUI Galway, Ireland

3. Archaeology and Palaeoecology, School of Natural and Built Environment, Queen’s University Belfast, Belfast, Northern Ireland

**Methods**

**Sampling and Sequencing Preparation**

The petrous bones of two individuals with Multiple Osteochondromas from Ballyhanna, Co. Donegal (Excavation number 03E1384) were sampled for DNA extraction and shotgun sequencing (Licence to Alter Number 6861, National Museum of Ireland).

Bones were photographed before modification, and exposed to UV light for 30 minutes on either side to remove surface contaminants. The surface of each bone was cleaned using a drill bit, and a triangular section of the otic capsule was cut. Half was stored as a bone fragment, and the other half was pulverised in a Mixer Mill 400 (Retsch). Extracts were prepared from 0.1g of this powder. One extract of Sk331 was prepared as in Boessenkool et al (2017)[(1)](https://paperpile.com/c/mGN1wR/LcGFS)⁠: bone powder was initially incubated in 0.5% bleach for 15 minutes to remove contaminants. After centrifuging for a few seconds at 13000 rpm, the bleach supernatant was discarded, and the remaining pellet was washed three times with UV water. The pellet was then incubated in UV-ed EDTA at 37°C for 30 minutes at 900rpm. This was then centrifuged at 13000 rpm for 10 minutes and the supernatant was removed and stored. The remaining pellet was incubated in UV-ed extraction buffer with proteinase K for 48 hours at 37°C and 900rpm. This was centrifuged at 13000 rpm for 10 minutes, and the supernatant was transferred to an Amicon Ultra-4 Centrifugal Filter unit 30kDa, diluted in 3mL 10 mM Tris-EDTA buffer and centrifuged at 5000 rpm until 100uL of solution remained. This volume was then added to a silica column (MinElute PCR purification kit, Qiagen, Hilden, Germany) and purified according to the manufacturer’s instructions. Sk197 was extracted following a similar protocol but with some modifications as in Dabney et al. (namely, shorter incubation with proteinase K (24hr), lower centrifugation speeds and the use of Zymo-Spin V columns (Zymo Research) rather than the Amicon columns)[(2)](https://paperpile.com/c/mGN1wR/04Rqt). An additional extract from the initial EDTA wash was prepared for Sk197.

3 sequencing libraries were prepared from 16.25uL of each extract (total: 9 libraries). Each of these were USER-treated to mitigate the effects of post-mortem damage. Libraries were prepared as in Meyer and Kircher (2010), with modifications as in Gamba et al (2014)[(3,4)](https://paperpile.com/c/mGN1wR/tyI9o+j8Ecx)⁠. Libraries and controls were amplified using Accuprime Pfx (Life technology); indexing primers p5 and p7; 10x reaction buffer and 3uL of library/water with a total reaction volume of 25uL. The first libraries for each extract were amplified for 14 cycles for an initial screen. The remaining PCRs were amplified for 10-13 cycles for high coverage sequencing. PCRs were purified with the MinElute PCR purification kit, and the concentrations of these products were quantified using the Agilent Tapestation system with a D1000 screentape (Sk331) or a QuBit with the High Sensitivity dsDNA assay (Invitrogen) (Sk197). Samples were screened and sequenced to a high read depth on a Novaseq 6000 platform using paired-end 50bp reads.

**Alignment and Data Processing**

The quality of demultiplexed fastq files was assessed using the FASTQC suite[(5)](https://paperpile.com/c/mGN1wR/IugGV). Adapter sequences were trimmed using AdapterRemoval v2.2.2 and paired end reads were collapsed [(6)](https://paperpile.com/c/mGN1wR/ydBvf). Trimmed reads were aligned to the hs37d5 reference genome using bwa aln with relaxed parameters and the seed disabled (-o 2 -n 0.01 -l 16500) [(7)](https://paperpile.com/c/mGN1wR/RvlmN). PCR and optical duplicates were removed using PicardTools v2.22.1 MarkDuplicates (http://broadinstitute.github.io/picard), and reads were filtered for mapping quality > 20 using samtools [(8)](https://paperpile.com/c/mGN1wR/ja5B4). Bam files were merged to sample level and indel realignment was performed using GATK⁠ [(9)](https://paperpile.com/c/mGN1wR/WjEQb). Reads were “softclipped” by reducing the quality score of the first and last two bases of each read to a PHRED score of two. Bam files were then fished for exact p7 index matches, as one base-pair mismatches had been allowed during demultiplexing.

Mitochondrial and X chromosome contamination estimates were used to assess authenticity of this data. Mitochondrial contamination was estimated using the frequency of heteroplasmic sites when aligned to the revised Cambridge Reference sequence, both including and excluding possible postmortem damage. X chromosome contamination levels were estimated using ANGSD as in Rasmussen et al. (2011) [(10)](https://paperpile.com/c/mGN1wR/6wva6).

**Uniparental Haplogroups**

Mitochondrial consensus sequences were estimated using samtools mpileup and vcfutils [(11)](https://paperpile.com/c/mGN1wR/tUMGE). Each site had a minimum read depth of 5 and base quality 30. Consensus fasta files were used as input to Mitomaster, which was used to estimate mitochondrial haplogroups [(12)](https://paperpile.com/c/mGN1wR/0kIDl).

Y haplogroups were investigated by calling representative SNPs of likely haplogroups from the International Society of Genetic Genealogy 2020 Y-DNA tree (v. 15.58) [(13)](https://paperpile.com/c/mGN1wR/m6epv). Base calls across these loci were generated for both samples using GATK’s pileup tool, and loci with derived alleles were flagged. These were then manually assessed to call Y haplogroups.

**Contamination Estimation**

Contamination levels were estimated using both mitochondrial and X chromosomal data. The method described in Rasmussen et al (2011) as implemented in ANGSD was used to estimate X contamination. Mitochondrial contamination was estimated by calculating the proportion of polymorphic sites with multiple alleles called. Estimates were also calculated for transversions only to mitigate the impact of any residual damage, but these estimates were very similar to those using all sites (**Table S3**).

**Pseudohaploid SNP calling**

GATK was used to pileup SNPs of interest for pseudohaploid analyses from the final BAM files (see **Table S6** for details). These sites were filtered for minimum base quality 30, and one read was sampled at random to generate pseudohaploid calls.

**Modern Population Affinities**

*Principal Components Analysis*

Principal components were calculated on a dataset of modern Northwestern Europeans (including Irish individuals) [(14–16)](https://paperpile.com/c/mGN1wR/dGoZj+YSFEy+jkv1C), and Sk331 and 197 were projected onto this using smartpca v8000 with shrinkmode:YES [(17,18)](https://paperpile.com/c/mGN1wR/GvpHZ+wVnVk). No outlier iterations were performed, and SNPs in LD were eliminated with an r^2^ threshold > 0.2. Modern Irish and European data were generated by third parties (see referenced publications) and are available from the European Genome-Phenome Archive under accession numbers EGAS00001002769, EGAD00010000632 and EGAD00000000120.

**Exome Scan**

A scan to search for possible dominant acting variants was developed. Bcftools v1.10.2 was used to call possible variant sites in autosomal exons from bam files aligned to hs37d5 [(11)](https://paperpile.com/c/mGN1wR/tUMGE), and calls were filtered for quality using the command

“bcftools mpileup -R ${bed} --count-orphans --annotate FORMAT/AD,INFO/AD --ignore-RG --fasta-ref ${ref} ${bamfile} -Ou | bcftools call -mv --format-fields GQ,GP -Ou | bcftools filter -s LowQual -Oz -o ${id}.exome.unsorted.vcf.gz”.

Co-ordinates of autosomal exons were downloaded from the UCSC table browser tool on 15-09-2020 in BED format using the NCBI RefSeq database, including 2bp up- and downstream of the coding sequences [(19)](https://paperpile.com/c/mGN1wR/5Kd5T).

SnpEff and SnpSift were used to annotate these possible variants in terms of molecular impact on canonical transcripts [(20,21)](https://paperpile.com/c/mGN1wR/QKvBF+jZgzO). Possible variants were filtered for minimum allelic depth 3 and maximum read depth less than twice the mean genomic coverage. A minimum genotyping quality of 50 was used. Sites were then filtered for high or moderate molecular impact, and variants in genes with a high probability of loss of function intolerance (pLI > 90%) according to gnomADv2.1.1 were retained [(22)](https://paperpile.com/c/mGN1wR/R1xAi). These variants were filtered for low (<1%) allele frequency in gnomad and predicted pathogenic effect by both SIFT and Polyphen2 (SNPs) or just SIFT (indels) [(23,24)](https://paperpile.com/c/mGN1wR/hIrSb+qngFI). The ACMG classification guidelines were used to assess support for the candidate pathogenic variants [(25)](https://paperpile.com/c/mGN1wR/qNyKs).

**Imputation**

Diploid genotypes were imputed for all autosomes to confirm that these individuals were unlikely to be related, as well as to visualise the haplotypes around the two genes associated with Multiple Osteochondromas (*EXT1* and *EXT2*) to confirm that a common mutation was unlikely to have been missed by the screening method described above.

Autosomal imputation was performed on both individuals using GLIMPSE[(26)](https://paperpile.com/c/mGN1wR/aUuM4). Genotype likelihoods for autosomal biallelic SNPs in the 1000 genomes project phase 3 panel were called for each individual using bcftools v1.10.2 as in the GLIMPSE documentation [(11,26)](https://paperpile.com/c/mGN1wR/tUMGE+aUuM4). Individuals were merged on a per-chromosome basis and imputed in chunks of 2Mb with a 200kB buffer region, using the Phase 3 panel of the 1000 genomes project as a reference dataset [(27)](https://paperpile.com/c/mGN1wR/S27G1). A genotype probability filter of 99% was used for all downstream analyses of this data.

This data was merged with a dataset of imputed genotypes for Iron Age and Medieval individuals from Europe and Asia[(28–36)](https://paperpile.com/c/mGN1wR/bwUqp+wYCX3+SzKaI+s5Fov+xRSc1+13PBL+9X9lC+XC706+oEBz2). These genotypes were imputed using the same method described above. Sites were filtered for a minor allele frequency of 5%, and individuals with genotype missingness > 10% were removed from the dataset. Only sites with zero missingness were retained for haplotype analysis, leaving a total of 2183372 SNPs for analysis. This final dataset was phased using SHAPEITv2 with the 1000 Genomes Project Phase 3 reference panel[(27,37)](https://paperpile.com/c/mGN1wR/lTQ6e+S27G1). These phased genotypes were used as input for IBD analysis and haplotype analysis around the disease genes.

**IBD Analysis**

Segments of the genome inherited from a common ancestor (IBD segments) were inferred in our phased dataset using the program RefinedIBD [(38)](https://paperpile.com/c/mGN1wR/bH70l). Default parameters (minimum length 1.5 cM; minimum LOD score 3) were used. Segments less than 0.6 cM apart with only one discordant genotype between them were subsequently merged using the program merge-ibd-segments.17Jan20.102.jar, as recommended by the authors of RefinedIBD. The total segment length between two individuals within different size bins (1.5-4cM; 4-10cM; 10-20cM; >20 cM) were investigated to confirm that the individuals from Ballyhanna do not share excessive IBD compared to a reference contemporaneous population.

**EXT1/2 Haplotypes**

Relationships between the haplotypes at known disease genes (*EXT1*; *EXT2)* in affected individuals was investigated in the context of a larger imputed dataset of published Iron Age and Historical samples [(28–36)](https://paperpile.com/c/mGN1wR/bwUqp+wYCX3+SzKaI+s5Fov+xRSc1+13PBL+9X9lC+XC706+oEBz2). The regions spanned by *EXT1* and *EXT2* exons and 50kB up- and down-stream were visualised using the tool haplostrips[(39)](https://paperpile.com/c/mGN1wR/KquPJ)⁠.

**Radiocarbon Dating**

Radiocarbon dating was undertaken on two rib samples. The dates were repeated to ensure accuracy.

| **Lab Code** | Sample/Context | Years BP | Calibrated Date Ranges |
| --- | --- | --- | --- |
| UBA-11442 | Rib from Sk197 | 1223 +/- 27 | AD 708-877 (1 σ); AD 689-885 (2 σ) |
| UBA-11442 Repeat |  | 1162 +/- 24 | AD 775-950 (1 σ); AD 774-975 (2 σ) |
| UBA-11443 | Rib from Sk331 | 856 +/- 24 | AD 1175-1221 (1 σ); AD 1158-1260 (2 σ) |
| UBA-11443 Repeat |  | 928 +/- 40 | AD 1042-1162 (1 σ); AD 1031-1211(2 σ) |

Calibration: Reimer et al, 2020 [(40)](https://paperpile.com/c/mGN1wR/LntRO)

**References**

1. [Boessenkool S, Hanghøj K, Nistelberger HM, Der Sarkissian C, Gondek AT, Orlando L, et al. Combining bleach and mild predigestion improves ancient DNA recovery from bones. Mol Ecol Resour. 2017;17(4):742–51.](http://paperpile.com/b/mGN1wR/LcGFS)

2. [Dabney J, Knapp M, Glocke I, Gansauge MT, Weihmann A, Nickel B, et al. Complete mitochondrial genome sequence of a Middle Pleistocene cave bear reconstructed from ultrashort DNA fragments. Proc Natl Acad Sci U S A. 2013;110(39):15758–63.](http://paperpile.com/b/mGN1wR/04Rqt)

3. [Meyer M, Kircher M. Illumina sequencing library preparation for highly multiplexed target capture and sequencing. Cold Spring Harb Protoc [Internet]. 2010;5(6). Available from:](http://paperpile.com/b/mGN1wR/tyI9o) <http://dx.doi.org/10.1101/pdb.prot5448>

4. [Gamba C, Jones ER, Teasdale MD, McLaughlin RL, Gonzalez-Fortes G, Mattiangeli V, et al. Genome flux and stasis in a five millennium transect of European prehistory. Nat Commun. 2014 Dec 21;5(1):5257.](http://paperpile.com/b/mGN1wR/j8Ecx)

5. [Andrews S. FastQC [Internet]. 2010. Available from:](http://paperpile.com/b/mGN1wR/IugGV) <https://www.bioinformatics.babraham.ac.uk/projects/fastqc/>

6. [Schubert M, Lindgreen S, Orlando L. AdapterRemoval v2: rapid adapter trimming, identification, and read merging. BMC Res Notes. 2016;9(1):88.](http://paperpile.com/b/mGN1wR/ydBvf)

7. [Li H, Durbin R. Fast and accurate short read alignment with Burrows-Wheeler transform. Bioinformatics. 2009 Jul 15;25(14):1754–60.](http://paperpile.com/b/mGN1wR/RvlmN)

8. [Li H, Handsaker B, Wysoker A, Fennell T, Ruan J, Homer N, et al. The Sequence Alignment/Map format and SAMtools. Bioinformatics. 2009 Aug 15;25(16):2078–9.](http://paperpile.com/b/mGN1wR/ja5B4)

9. [McKenna A, Hanna M, Banks E, Sivachenko A, Cibulskis K, Kernytsky A, et al. The Genome Analysis Toolkit: A MapReduce framework for analyzing next-generation DNA sequencing data. Genome Res. 2010 Sep 1;20(9):1297–303.](http://paperpile.com/b/mGN1wR/WjEQb)

10. [Rasmussen M, Guo X, Wang Y, Lohmueller KE, Rasmussen S, Albrechtsen A, et al. An Aboriginal Australian genome reveals separate human dispersals into Asia. Science. 2011 Oct;334(6052):94–8.](http://paperpile.com/b/mGN1wR/6wva6)

11. [Li H. A statistical framework for SNP calling, mutation discovery, association mapping and population genetical parameter estimation from sequencing data. Bioinformatics. 2011 Nov 1;27(21):2987–93.](http://paperpile.com/b/mGN1wR/tUMGE)

12. [Lott MT, Leipzig JN, Derbeneva O, Xie HM, Chalkia D, Sarmady M, et al. mtDNA Variation and Analysis Using Mitomap and Mitomaster. Curr Protoc Bioinformatics. 2013 Dec;44(123):1.23.1–1.23.26.](http://paperpile.com/b/mGN1wR/0kIDl)

13. [International Society of Genetic Genealogy. Y-DNA Haplogroup Tree 2020, Version: 15.58, Date: 21 May 2020 [Internet]. 2020. Available from:](http://paperpile.com/b/mGN1wR/m6epv) <http://www.isogg.org/tree/>

14. [Byrne RP, Martiniano R, Cassidy LM, Carrigan M, Hellenthal G, Hardiman O, et al. Insular Celtic population structure and genomic footprints of migration. Falush D, editor. PLoS Genet. 2018 Jan 25;14(1):e1007152.](http://paperpile.com/b/mGN1wR/dGoZj)

15. [Leslie S, Winney B, Hellenthal G, Davison D, Boumertit A, Day T, et al. The fine-scale genetic structure of the British population. Nature. 2015 Mar 19;519(7543):309–14.](http://paperpile.com/b/mGN1wR/YSFEy)

16. [International Multiple Sclerosis Genetics Consortium, Wellcome Trust Case Control Consortium 2, Sawcer S, Hellenthal G, Pirinen M, Spencer CCA, et al. Genetic risk and a primary role for cell-mediated immune mechanisms in multiple sclerosis. Nature. 2011 Aug 10;476(7359):214–9.](http://paperpile.com/b/mGN1wR/jkv1C)

17. [Patterson N, Price AL, Reich D. Population Structure and Eigenanalysis. PLoS Genet. 2006;2(12):e190.](http://paperpile.com/b/mGN1wR/GvpHZ)

18. [Price AL, Patterson NJ, Plenge RM, Weinblatt ME, Shadick NA, Reich D. Principal components analysis corrects for stratification in genome-wide association studies. Nat Genet. 2006 Aug 23;38(8):904–9.](http://paperpile.com/b/mGN1wR/wVnVk)

19. [Karolchik D, Hinrichs AS, Furey TS, Roskin KM, Sugnet CW, Haussler D, et al. The UCSC Table Browser data retrieval tool. Nucleic Acids Res. 2004 Jan 1;32(suppl_1):D493–6.](http://paperpile.com/b/mGN1wR/5Kd5T)

20. [Cingolani P, Platts A, Wang LL, Coon M, Nguyen T, Wang L, et al. A program for annotating and predicting the effects of single nucleotide polymorphisms, SnpEff: SNPs in the genome of Drosophila melanogaster strain w1118; iso-2; iso-3. Fly . 2012;6(2):80–92.](http://paperpile.com/b/mGN1wR/QKvBF)

21. [Cingolani P, Patel VM, Coon M, Nguyen T, Land SJ, Ruden DM, et al. Using Drosophila melanogaster as a Model for Genotoxic Chemical Mutational Studies with a New Program, SnpSift. Front Genet. 2012;3:35.](http://paperpile.com/b/mGN1wR/jZgzO)

22. [Karczewski KJ, Francioli LC, Tiao G, Cummings BB, Alföldi J, Wang Q, et al. The mutational constraint spectrum quantified from variation in 141,456 humans. Nature. 2020;581(7809):434–43.](http://paperpile.com/b/mGN1wR/R1xAi)

23. [Kumar P, Henikoff S, Ng PC. Predicting the effects of coding non-synonymous variants on protein function using the SIFT algorithm. Nat Protoc. 2009;4(7):1073–82.](http://paperpile.com/b/mGN1wR/hIrSb)

24. [Adzhubei IA, Schmidt S, Peshkin L, Ramensky VE, Gerasimova A, Bork P, et al. A method and server for predicting damaging missense mutations. Nat Methods. 2010;7(4):248–9.](http://paperpile.com/b/mGN1wR/qngFI)

25. [Richards S, Aziz N, Bale S, Bick D, Das S, Gastier-Foster J, et al. Standards and guidelines for the interpretation of sequence variants: A joint consensus recommendation of the American College of Medical Genetics and Genomics and the Association for Molecular Pathology. Genet Med. 2015;17(5):405–24.](http://paperpile.com/b/mGN1wR/qNyKs)

26. [Rubinacci S, Ribeiro DM, Hofmeister RJ, Delaneau O. Efficient phasing and imputation of low-coverage sequencing data using large reference panels. Nat Genet. 2021;53(1):120–6.](http://paperpile.com/b/mGN1wR/aUuM4)

27. [The 1000 Genomes Project Consortium, Gibbs RA, Boerwinkle E, Doddapaneni H, Han Y, Korchina V, et al. A global reference for human genetic variation. Nature. 2015 Oct 1;526(7571):68–74.](http://paperpile.com/b/mGN1wR/S27G1)

28. [Amorim CEG, Vai S, Posth C, Modi A, Koncz I, Hakenbeck S, et al. Understanding 6th-century barbarian social organization and migration through paleogenomics. Nat Commun. 2018 Dec 11;9(1):3547.](http://paperpile.com/b/mGN1wR/bwUqp)

29. [Brunel S, Bennett EA, Cardin L, Garraud D, Barrand Emam H, Beylier A, et al. Ancient genomes from present-day France unveil 7,000 years of its demographic history. Proc Natl Acad Sci U S A. 2020;2020:1–8.](http://paperpile.com/b/mGN1wR/wYCX3)

30. [Damgaard P de B, Marchi N, Rasmussen S, Peyrot M, Renaud G, Korneliussen T, et al. 137 ancient human genomes from across the Eurasian steppes. Nature. 2018 May;557(7705):369–74.](http://paperpile.com/b/mGN1wR/SzKaI)

31. [Ferrando-Bernal M, Morcillo-Suarez C, de-Dios T, Gelabert P, Civit S, Díaz-Carvajal A, et al. Mapping co-ancestry connections between the genome of a Medieval individual and modern Europeans. Sci Rep. 2020 Apr 22;10(1):6843.](http://paperpile.com/b/mGN1wR/s5Fov)

32. [Kivisild T, Saag L, Hui R, Biagini SA, Pankratov V, D’Atanasio E, et al. Patterns of genetic connectedness between modern and medieval Estonian genomes reveal the origins of a major ancestry component of the Finnish population. Am J Hum Genet. 2021 Sep 2;108(9):1792–806.](http://paperpile.com/b/mGN1wR/xRSc1)

33. [Rodríguez-Varela R, Günther T, Krzewińska M, Storå J, Gillingwater TH, MacCallum M, et al. Genomic Analyses of Pre-European Conquest Human Remains from the Canary Islands Reveal Close Affinity to Modern North Africans. Curr Biol. 2017 Nov 6;27(21):3396–402.e5.](http://paperpile.com/b/mGN1wR/13PBL)

34. [Saag L, Laneman M, Varul L, Malve M, Valk H, Razzak MA, et al. The Arrival of Siberian Ancestry Connecting the Eastern Baltic to Uralic Speakers Further East. Curr Biol. 2019;29(10):1701–11.](http://paperpile.com/b/mGN1wR/9X9lC)

35. [Schiffels S, Haak W, Paajanen P, Llamas B, Popescu E, Loe L, et al. Iron Age and Anglo-Saxon genomes from East England reveal British migration history. Nat Commun. 2016 Dec 19;7(1):10408.](http://paperpile.com/b/mGN1wR/XC706)

36. [Veeramah KR, Rott A, Groß M, van Dorp L, López S, Kirsanow K, et al. Population genomic analysis of elongated skulls reveals extensive female-biased immigration in Early Medieval Bavaria. Proc Natl Acad Sci U S A. 2018 Mar 27;115(13):3494–9.](http://paperpile.com/b/mGN1wR/oEBz2)

37. [O’Connell J, Gurdasani D, Delaneau O, Pirastu N, Ulivi S, Cocca M, et al. A General Approach for Haplotype Phasing across the Full Spectrum of Relatedness. PLoS Genet. 2014;10(4):e1004234.](http://paperpile.com/b/mGN1wR/lTQ6e)

38. [Browning BL, Browning SR. Improving the accuracy and efficiency of identity-by-descent detection in population data. Genetics. 2013;194(2):459–71.](http://paperpile.com/b/mGN1wR/bH70l)

39. [Marnetto D, Huerta-Sánchez E. Haplostrips: revealing population structure through haplotype visualization. Methods Ecol Evol. 2017;8(10):1389–92.](http://paperpile.com/b/mGN1wR/KquPJ)

40. [Reimer PJ, Austin WEN, Bard E, Bayliss A, Blackwell PG, Ramsey CB, et al. The IntCal20 Northern Hemisphere Radiocarbon Age Calibration Curve (0–55 cal kBP). Radiocarbon. 2020 Aug;62(4):725–57.](http://paperpile.com/b/mGN1wR/LntRO)

**Supplementary figures**
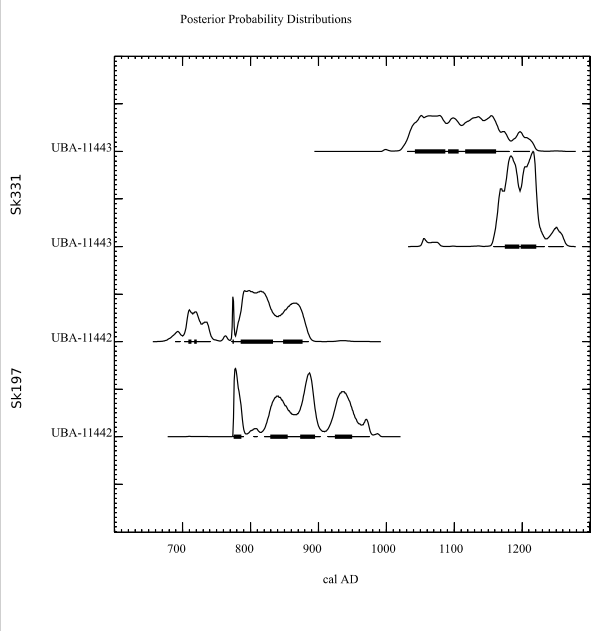


**Figure S1. Radiocarbon dates for Sk197 and Sk331.** Posterior distribution of estimated age is displayed, where the x-axis is in calendar years AD. UBA-11442 is Sk197 and UBA-11443 is Sk331.

**
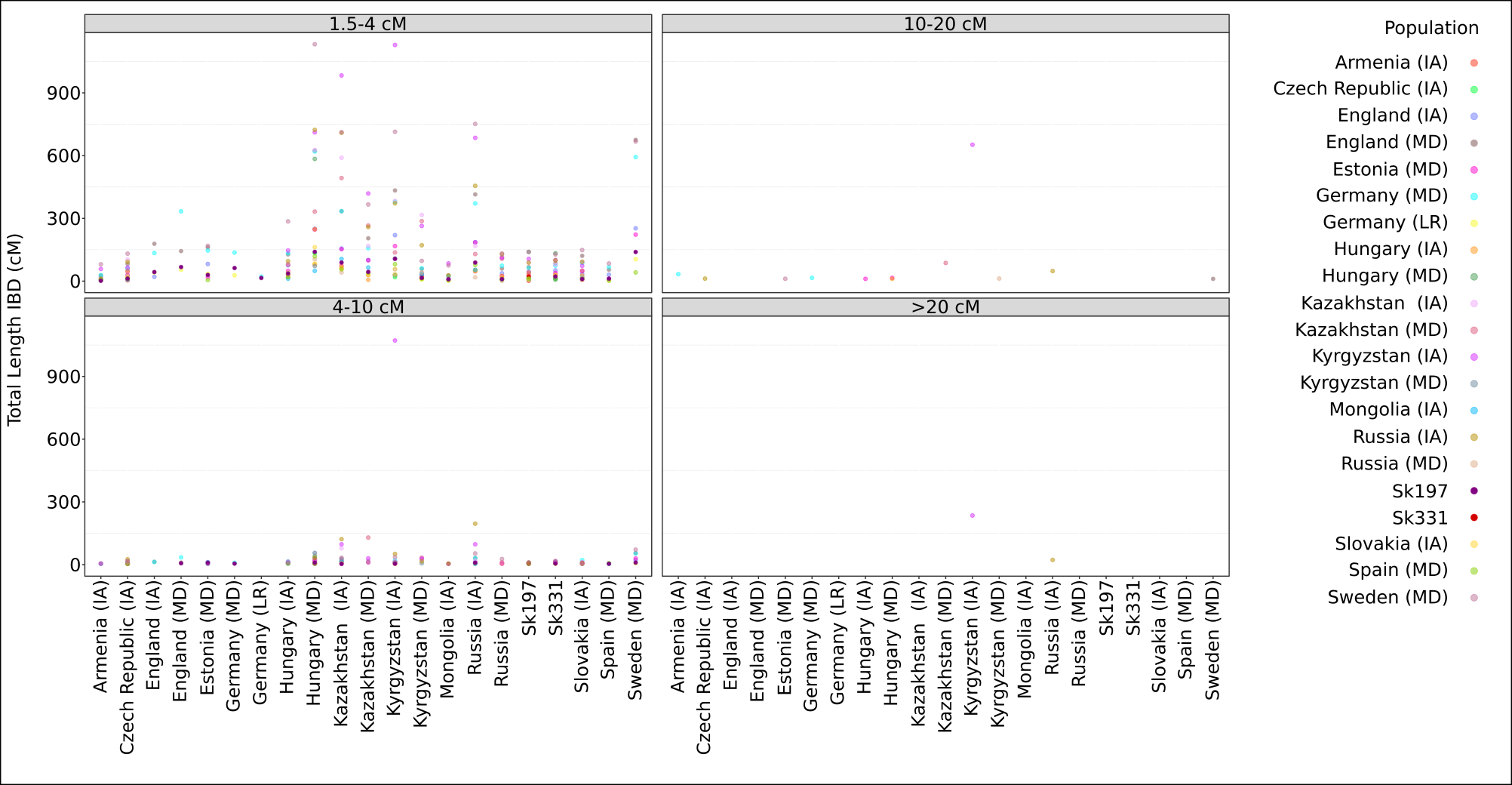
**

**Figure S2. IBD sharing in Iron Age and Medieval Eurasia.** Total Length of IBD shared between individuals within phased dataset, binned by size of segment, date and country. IA: Iron Age; LR: Late Roman; MD: Medieval. This dataset includes some pairs of relatives from Iron age Kyrgyzstan.

**Figure S3. Haplotype structure around *EXT1* and *EXT2*.** Haplotypes 50kB up- and down-stream of the genes *EXT1* and *EXT2*
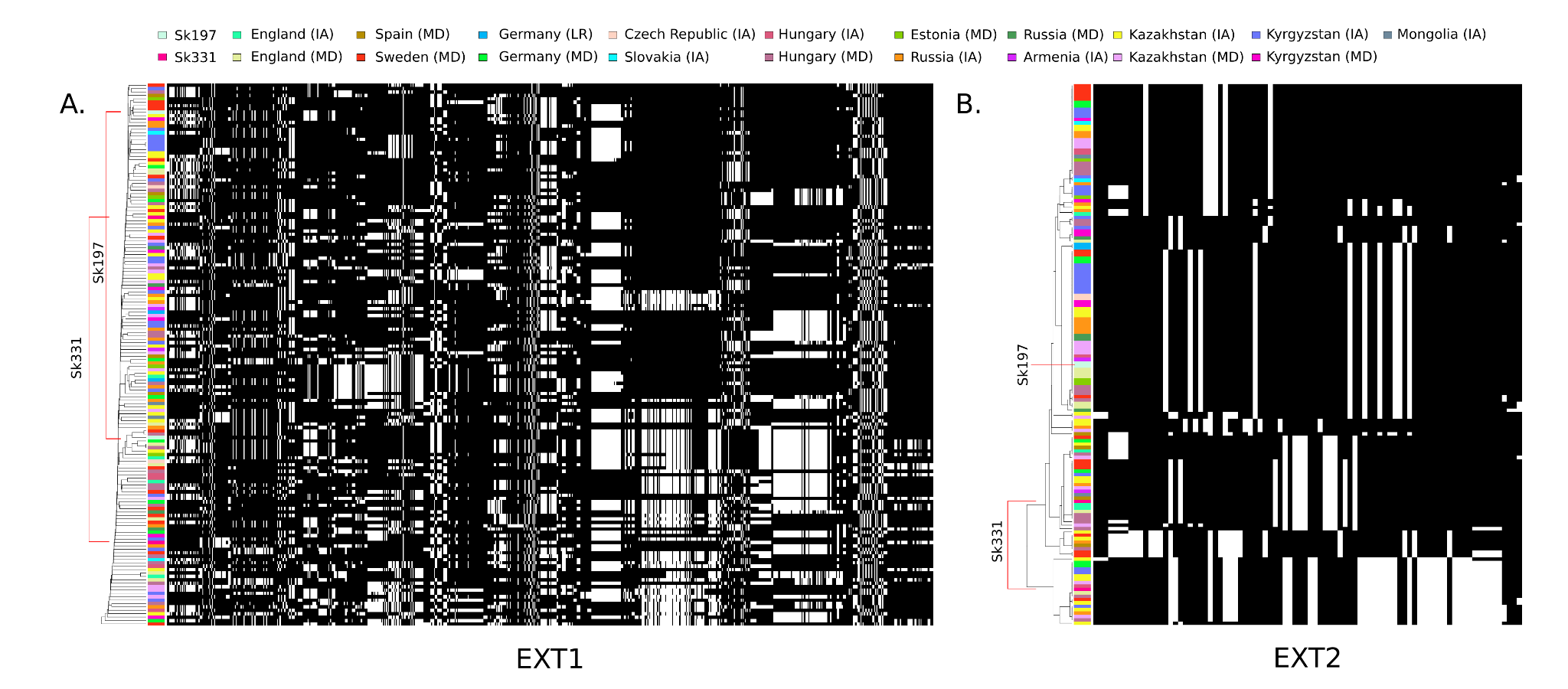
were visualised. Haplotypes corresponding to Sk197 and Sk331 are marked. Black: Reference Allele; White: Alternate allele; IA: Iron Age; LR: Late Roman; MD: Medieval.
